# Network-based discovery of regulatory drivers of cognitive decline in alzheimer’s disease

**DOI:** 10.1038/s41514-026-00443-0

**Published:** 2026-07-16

**Authors:** Danish Anwer, Arina A, Agata Marchi, Eduard Kerkhoven, Annikka Polster

**Affiliations:** 1 Division of Systems and Synthetic Biology, Department of Life Sciences, Chalmers University of Technology, Gothenburg, Sweden; 2 Department of Microbiology, Oslo University Hospital and University of Oslo, Oslo, Norway

**Keywords:** Neuroscience, Diseases

## Abstract

Alzheimer’s disease (AD) is a multifactorial neurodegenerative disorder marked by progressive cognitive decline, yet its transcriptional regulatory architecture remains poorly understood. Here, we model sample-specific gene regulatory networks (GRNs) from dorsolateral prefrontal cortex transcriptomes of 87 individuals with AD and 67 non-cognitively impaired (NCI) controls and use a machine learning classifier to detect consistent disease-specific network features. This sample-specific network approach captures inter-individual variation in transcriptional regulation and revealed 22 key transcription factor-gene regulations that distinguish AD from NCI with 96% weighted accuracy. The key transcription factor-gene interactions were enriched in pathways central to AD pathology, including synaptic signalling, mitochondrial function, proteostasis, and neuroinflammation. Network analysis uncovered significant differences in regulatory connectivity between AD and controls, with ZNF225, ZNF849, and ZNF548 emerging as AD-specific regulatory hubs. Moreover, several key regulatory edges showed significant correlations with longitudinal cognitive decline, supporting their clinical relevance. Our findings highlight pervasive transcriptional dysregulation in AD, emphasizing sample-specific GRN modelling’s value in uncovering regulatory mechanisms.

## Introduction

Alzheimer’s disease (AD) is the most prevalent form of dementia, characterized by progressive cognitive decline, memory impairment, and eventual loss of autonomy and death. With over 50 million people currently affected worldwide, and prevalence rising due to increasing life expectancy^1^, AD represents a significant global health burden. In addition to its personal and societal costs, the disease remains without a cure or effective preventive intervention, underscoring the urgent need to understand its underlying molecular mechanisms^2^.

Although the hallmark pathological features, amyloid-β plaques and neurofibrillary tangles, have been studied for decades^3^, the mechanisms underlying AD pathophysiology and progression remain incompletely understood. Genome-wide association studies (GWAS) have identified over 400 AD-associated loci^4^, yet these variants typically exhibit small effect sizes and collectively account for only a modest portion of heritability^5^. This gap, often referred to as “missing heritability”, suggests that complex, systems-level interactions between genes may play a larger role in AD pathogenesis than previously appreciated^6^. Recent conceptual frameworks, such as the omnigenic model^7^, propose that the functional impact of disease-associated variation is distributed across vast gene regulatory networks (GRNs), in which both core and peripheral genes contribute to disease through coordinated transcriptional regulation. In this view, transcriptional dysregulation in disease-relevant tissues, in this case the brain, is likely mediated not by isolated genes, but by disruptions in tissue-specific regulatory networks that govern key cellular processes.

GRN-based approaches thus offer a promising systems-level lens through which to investigate AD. Unlike differential expression analyses that identify gene-level changes, GRNs provide insight into regulatory logic: how transcription factors (TFs) control gene expression and how those interactions change in disease^7^. Prior studies have demonstrated the relevance of GRNs in neurodegenerative disorders, but most analyses to date rely on aggregated population-level data^8,9^. Such approaches overlook inter-individual variation, a critical limitation in AD, where clinical and molecular heterogeneity is important.

In this study, we applied a sample-specific network modelling strategy to reconstruct individual gene regulatory networks from postmortem dorsolateral prefrontal cortex (DLPFC) transcriptomes of individuals with and without AD. Using PANDA^10^ and LIONESS^11^ algorithms, we reconstructed individual TF-target gene networks for each study participant and analysed them with machine learning feature selection to identify a minimal yet informative set of regulatory interactions that distinguish AD from non-cognitively impaired (NCI) individuals. By focusing on transcriptional regulation rather than individual gene expression alone, we aim to reveal clinically relevant network-level mechanisms that underpin disease pathophysiology.

Taken together, we aim to define core regulatory modules and transcriptional hubs that are consistently perturbed in AD, providing a systems-level understanding of disease mechanisms.

## Results

### Cohort selection for RNA-Seq analysis

The study included 633 participants with RNA-Seq data for the discovery dataset. Participants were categorized based on cognitive diagnosis (cogdx) as follows: 201 with no cognitive impairment (NCI), 220 with Alzheimer’s disease (AD), 33 with AD and additional cause of cognitive impairment (CI), 11 with other forms of dementia, 158 with mild cognitive impairment (MCI) without another cause of CI, and 10 with MCI and another cause of CI. Neuropathological classification (CERAD) further categorized participants as 184 definite AD, 218 probable AD, 67 possible AD, and 164 with no AD. In this study, to maximize diagnostic specificity and reduce potential confounding, AD cases and NCI controls were defined using the combined Cogdx and CERAD criteria described in Methods. After applying these criteria, the final discovery cohort included 110 AD cases and 78 NCI controls. For the validation dataset, we applied similar criteria, resulting in 16 AD cases and 23 NCI controls for downstream analysis.

### Preprocessed RNA-Seq expression for gene regulatory network construction

After applying the CPM threshold filter to the 110 AD and 78 NCI samples, the dataset was refined to include 17,330 genes that met the expression criteria, retaining genes that were expressed in at least 50% of samples in either the AD or the NCI group. To address potential batch effects arising from RNA-sequencing batches (i.e., sequencing runs), we applied ComBat-Seq^12^ a method specifically designed for count-based RNA-Seq data. Following this correction, we observed that batches 0 and 7 still had a significant impact on the expression counts. We first removed batch 7, which left 97 AD and 73 NCI samples. Next, we removed batch 0, resulting in 94 AD and 70 NCI samples. Finally, we applied quantile-based thresholds (5th and 95th percentiles) to filter out extreme samples based on their principal component analysis (PCA) components. After removing outliers, 87 AD and 67 NCI samples were retained for downstream analysis. PCA plots illustrating the effect of each filtering step are shown in Supplementary Fig. S1. The distribution of samples across sequencing batches is summarized in Supplementary Data S2, and the batch distribution histogram before and after filtering is illustrated in Supplementary Fig. S2.

### Demographic and clinical characteristics

Participants with AD (*n* = 87) were marginally older than those with NCI (*n* = 67), with mean ages of 89.8 years (SD = 5.6) and 84.3 years (SD = 7.2), respectively, and an independent t-test revealed a significant age difference (*p*-value = 1.11e−06). Among AD cases, 56 were female, and 31 were male, while in the NCI group, 36 were female and 31 were male. A Chi-square test indicated no significant difference in gender distribution between the two groups (χ2 = 1.37, *p* = 0.24). APOE genotype distribution in the NCI group was as follows: ε2/ε2 (*n* = 1), ε2/ε3 (*n* = 15), ε2/ε4 (*n* = 1), ε3/ε3 (*n* = 47), and ε3/ε4 (*n* = 3). In the AD group, the APOE genotype distribution was ε2/ε2 (*n* = 4), ε2/ε3 (*n* = 3), ε2/ε4 (*n* = 42), ε3/ε3 (*n* = 37), and ε3/ε4 (*n* = 1). A Chi-square test comparing APOE genotypes between the AD and NCI groups revealed a significant difference in APOE genotype distribution (*p* = 5.01e−10).

The global AD pathology burden (gpath), a quantitative measure summarizing AD pathology, was significantly higher (t-test, *p*-value = 8.12e−38) in AD cases (mean = 1.48, SD = 0.59) compared to NCI controls (mean = 0.11, SD = 0.14). Gpath is derived from counts of three AD pathologies, neuritic plaques (n), diffuse plaques (d), and neurofibrillary tangles (NFT), assessed through microscopic examination of silver-stained slides from five brain regions: midfrontal cortex, midtemporal cortex, inferior parietal cortex, entorhinal cortex, and hippocampus (CA1)^13^.

### Gene regulatory network reconstruction and key features

Using PANDA and LIONESS, we reconstructed aggregate and sample-specific GRNs for AD and NCI. To focus on the most robust regulatory interactions, we retained the top 5% of edges based on the weights in aggregated AD and NCI GRNs (thresholds: 3.71 for AD, 3.67 for NCI). The cumulative distribution function (CDF) of edge weights, shown in Figure S3 in the supplementary material, illustrates these thresholds and the distribution of TF-gene edge strengths. After filtering, the networks contained 118,489 edges common to both conditions and 62,731 condition-specific edges. We retained both sets of edges for downstream analyses. We further validated that the top-weighted regulatory edges of sample-specific GRNs for AD and NCI largely correspond to known binding motifs in the regulatory regions of their target genes to confirm that the strongest predicted regulatory interactions are biologically plausible.

### Network metrics differences in AD-specific and NCI-specific GRNs

To identify potential differences in structural properties between AD and NCI regulatory networks, we performed network analysis on 87 AD and 67 NCI sample-specific GRNs. Our analysis, using the non-parametric Mann-Whitney U test, did not reveal a significant difference in density between AD-specific and NCI-specific GRNs (*p*-value = 0.19). Similarly, no significant differences were observed in the average degree (*p*-value = 0.17) or modularity (*p*-value = 0.10). However, we identified a notable difference in the largest connected component size (*p*-value = 0.025), with AD showing a noticeably smaller largest connected component than NCI, indicating a structural variation in network connectivity. This variation may reflect changes in central regulatory modules and thus key biological processes within the network. Additionally, nestedness showed a significant difference (*p*-value < 0.05), with NCI GRNs having higher nestedness than AD, highlighting variations in the connectivity patterns between nodes of different degrees. These findings highlight key network properties that may be altered in the context of AD. These results are summarized in Table 1, with box plots illustrating the distribution of network metrics across AD and NCI samples provided in Supplementary Fig. S4.

**Table 1:** AD and NCI GRNs structural features

Metric	AD (Mean ± Std)	NCI (Mean ± Std)	*P*-value
**Density**	0.51 ± 0.01	0.51 ± 0.008	0.19
**Average Weight Degree**	802.67 ± 332.4	805.91 ± 239.5	0.17
**Modularity**	0.21 ± 0.03	0.22 ± 0.03	0.1
**Giant Component Size**	17188.10 ± 23.51	17192.19 ± 23.03	0.025
**Nestedness**	-0.52 ± 0.04	-0.50 ± 0.04	0.046

### Sample-specific gene regulatory networks reveal key regulatory differences in AD

**Fig. 1:**
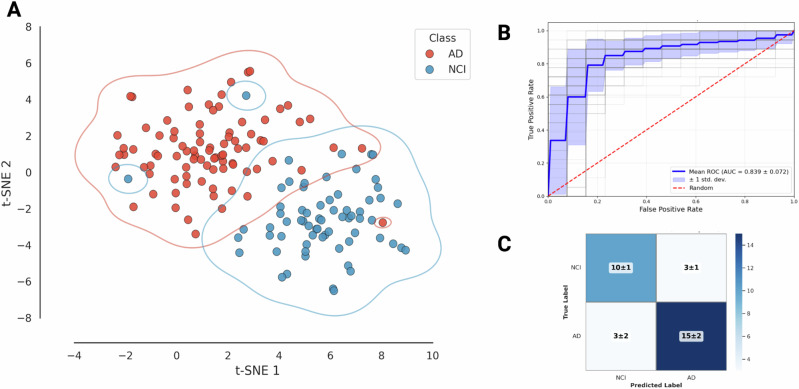
Results of Logistic Regression for classifying Alzheimer’s Disease (AD) and No Cognitive Impairment (NCI) Gene Regulatory Networks (GRNs) on Discovery Dataset. **A** t-SNE plot showing the separation of Alzheimer’s Disease (AD) and No Cognitive Impairment (NCI) Gene Regulatory Networks (GRNs) based on 22 identified GRN edges from Logistic Regression with Recursive Feature Elimination (RFE). **B** Receiver Operating Characteristic (ROC) curve illustrating the classification performance of Logistic Regression (AUC) over 100 Monte Carlo iterations. **C** Confusion matrix summarizing the average classification accuracy of Logistic Regression for AD and NCI GRNs over 100 Monte Carlo iterations.

**Fig. 2:**
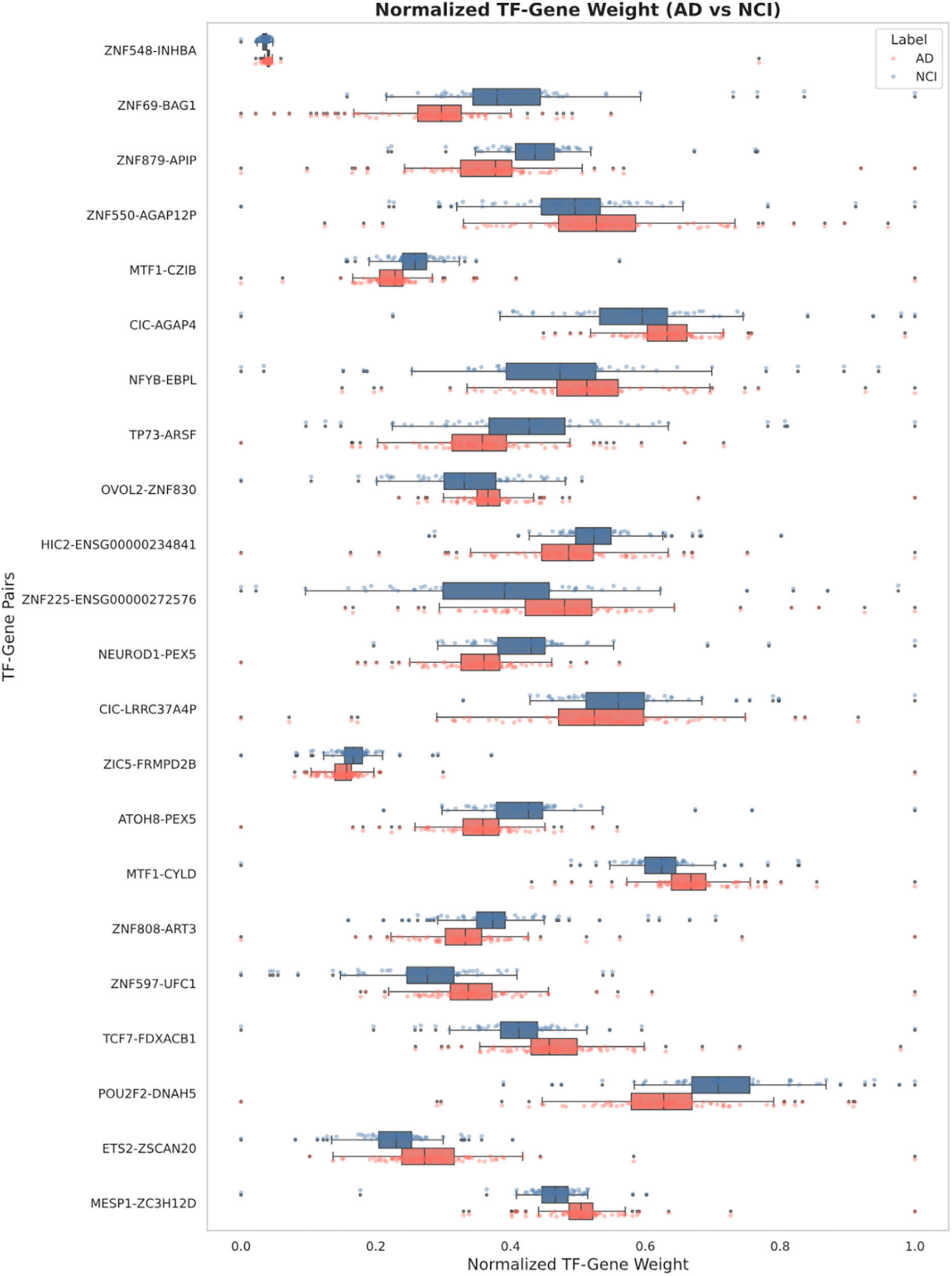
Box plot illustrating the distribution of TF-gene edge weights present in the 22 identified TF-gene edges for the No Cognitive Impairment (NCI) and Alzheimer’s Disease (AD) groups.

To identify key regulatory differences, we applied logistic regression with RFE on 118,489 high-confidence regulatory interactions, achieving a weighted classification accuracy of 96% based on TF-gene edge weights and an AUC of 0.936 on an independent test set. RFE identified 22 key TF-gene interactions that effectively discriminate AD from NCI. Model robustness was evaluated using Monte Carlo cross-validation with 100 iterations, yielding a weighted accuracy of 96 ± 0.2%, F1-score of 0.95 ± 0.5, precision of 0.963 ± 0.040, recall of 0.934 ± 0.060 and an AUC score of 0.839 ± 0.072. A confusion matrix in Fig. 1C illustrates the classification performance of Monte Carlo cross-validation on the discovery dataset, while t-SNE plots of the 22 key TF-target gene interactions demonstrating separation between AD and NCI samples are shown in Fig. 1A. Figure 1B shows the ROC curve for Logistic Regression based on 100 Monte Carlo iterations on the discovery dataset. Box plots of these interactions in Fig. 2 illustrate consistent differences between AD and NCI samples, highlighting common regulatory patterns across sample-specific GRNs. Bootstrapping analysis, in which random TF-target gene pairs were selected, demonstrated that the 96% weighted accuracy observed using the 22 key interactions was statistically significant (*p*-value < 0.05), indicating that this high classification performance was unlikely to occur by chance, as shown in supplementary Fig. S5.

**Fig. 3:**
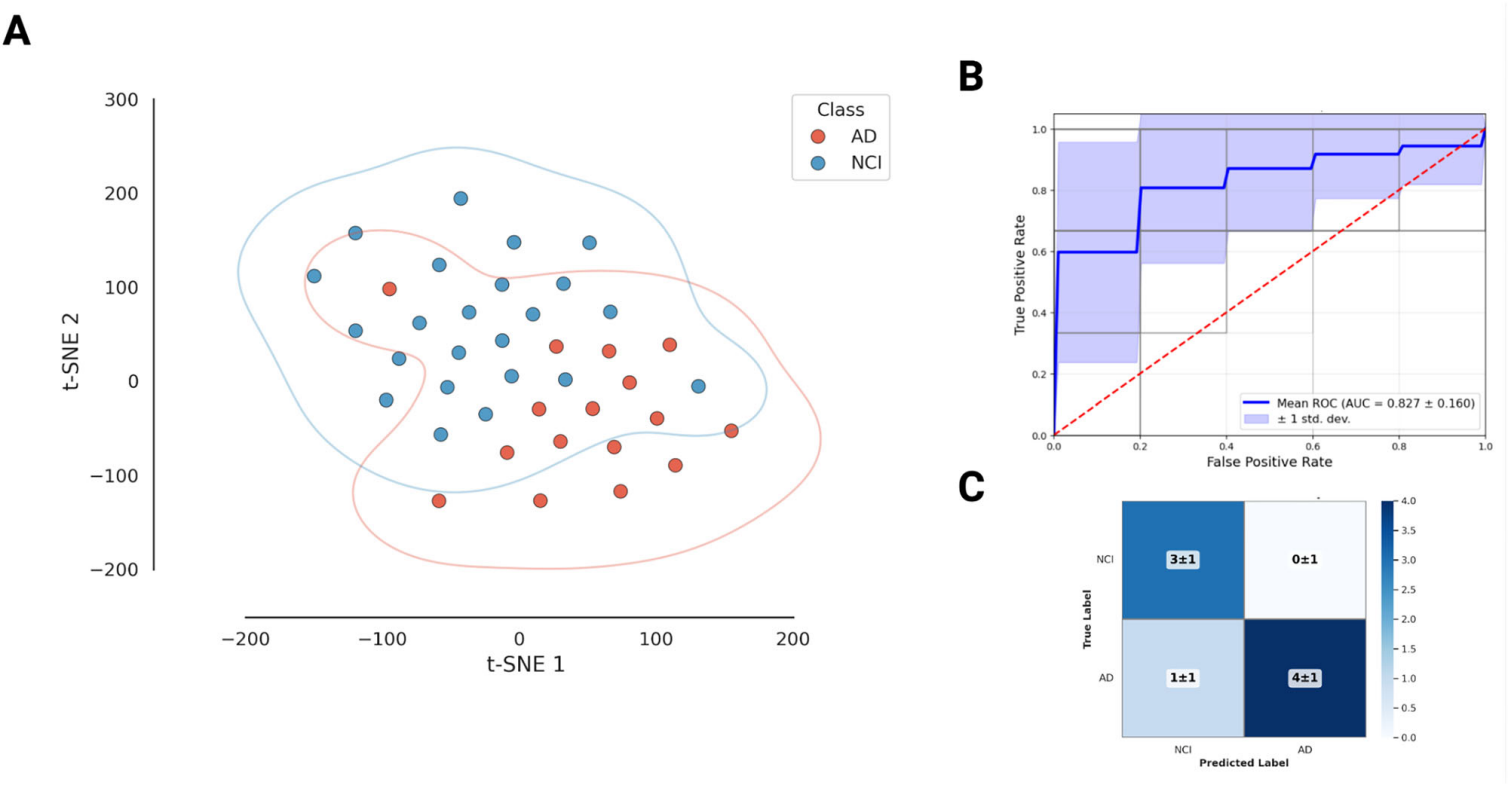
Results of Logistic Regression for classifying Alzheimer’s Disease (AD) and No Cognitive Impairment (NCI) Gene Regulatory Networks (GRNs) on Validation Independent Dataset. **A** t-SNE plot showing the separation of Alzheimer’s Disease (AD) and No Cognitive Impairment (NCI) Gene Regulatory Networks (GRNs) based on 22 identified GRN edges from Logistic Regression with Recursive Feature Elimination (RFE). **B** Receiver Operating Characteristic (ROC) curve illustrating the classification performance of Logistic Regression (AUC) over 100 Monte Carlo iterations. **C** Confusion matrix illustrating the performance of Logistic Regression in classifying Alzheimer’s Disease (AD) and No Cognitive Impairment (NCI) GRNs over 100 Monte Carlo iterations.

On the validation dataset, the model achieved a weighted accuracy of 0.787 ± 0.124, F1-score of 0.742 ± 0.140, precision of 0.687 ± 0.191, recall of 0.870 ± 0.188, and an AUC of 0.827 ± 0.160. A confusion matrix in Fig. 3C illustrates the classification performance of Monte Carlo cross-validation on the validation dataset, while t-SNE plots of the 22 key TF-target gene interactions demonstrating separation between AD and NCI samples are shown in Fig. 3A. Figure 3B shows the ROC curve for Logistic Regression based on 100 Monte Carlo iterations on the validation dataset.

**Fig. 4:**
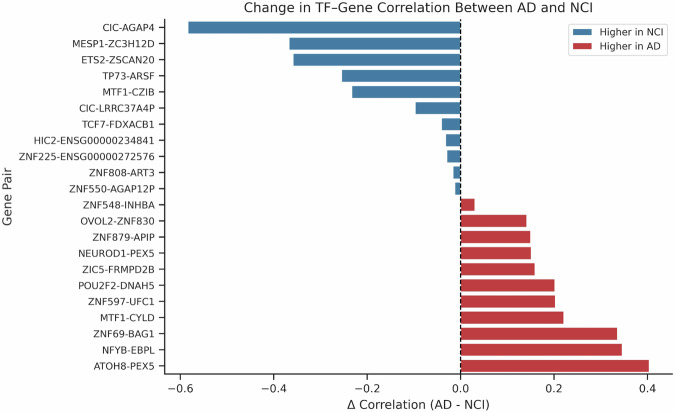
Differential Co-expression of Key TF-target gene pairs in Alzheimer’s disease.

We examined 22 key TF-target gene co-expression patterns in AD versus NCI samples in the discovery dataset and observed clear differences between the groups, as shown in Fig. 4, suggesting differential regulatory relationships between the conditions.

We also performed analyses using the condition-specific 62,731 edges, i.e., TF-gene interactions present only in either the AD or NCI GRNs. Across the 100 random train-test splits, classification performance based on these condition-specific edges was slightly lower than using the 22 key edges, with mean accuracy of 0.8315 ± 0.0613, mean F1-score of 0.7975 ± 0.0803, and mean AUC of 0.8631 ± 0.0720. This indicates that condition-specific interactions are less effective at distinguishing AD from NCI. Based on these results, we considered the common edges to provide a stronger and more reliable signal for differentiating the two conditions, and limited further analyses to these.

### Identification of hub genes

To determine whether any of the 22 identified TF-gene interactions represented highly influential regulators, we assessed which transcription factors functioned as regulatory hubs within the network. We identified three transcription factors, namely ZNF225, ZNF879, and ZNF548, as hub nodes within the GRNs among the 22 key TF-gene interactions. These hubs form key regulatory pairs with ENSG00000272576 (Lnc-DCUN1D4-1), APIP, and INHBA, respectively, within the identified 22 TF-gene interactions. In the GRNs of the validation AD dataset, two of the three hub transcription factors identified in the discovery set (ZNF225 and ZNF548) were confirmed, supporting their role as central regulators within the network with relevance in AD pathogenesis.

### Identification of dysregulated pathways in AD through Gene Set Enrichment Analysis

**Fig. 5:**
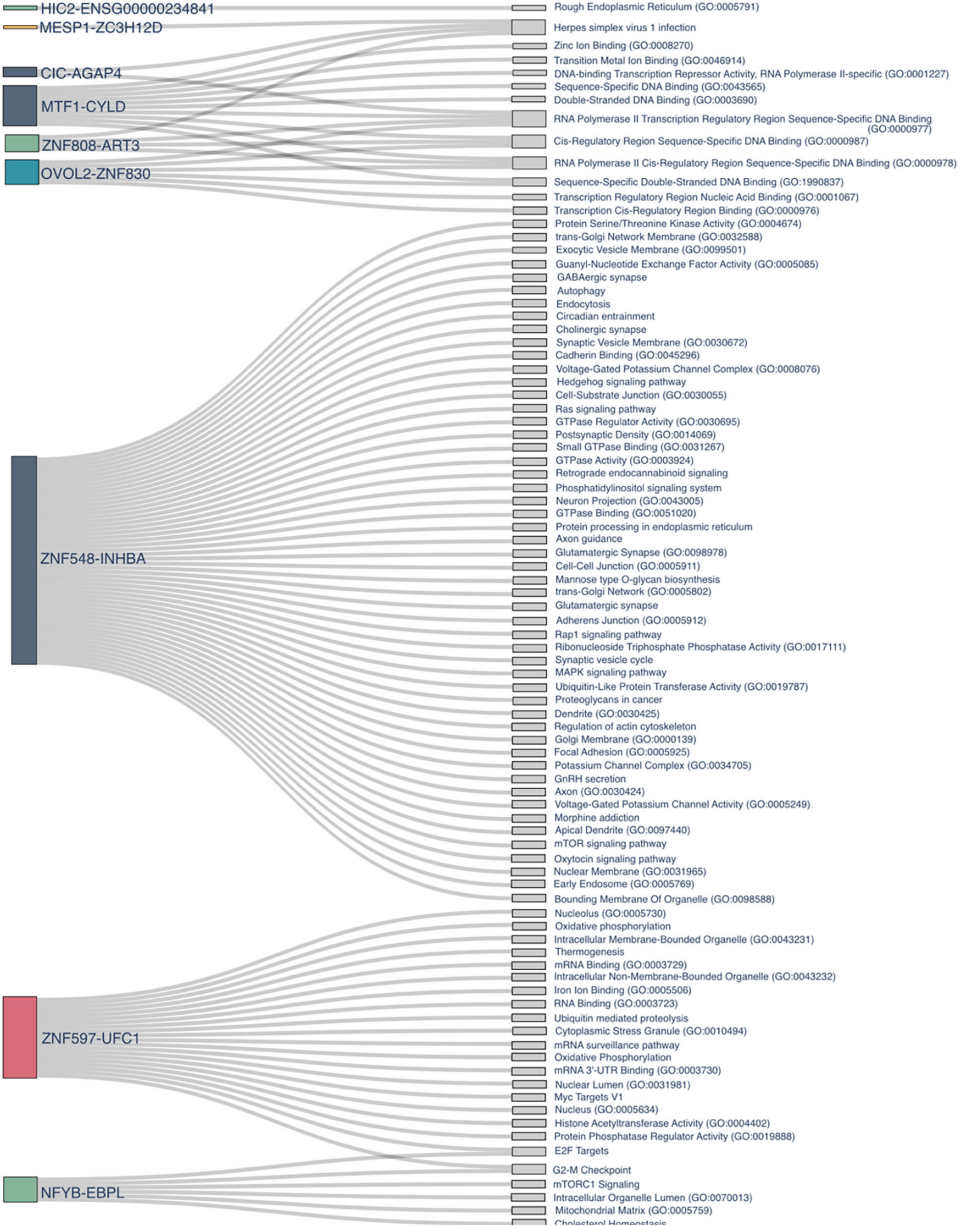
Sankey Plot of TF-Gene Regulatory Interactions and enriched pathways. The Sankey plot illustrates the regulatory flow from transcription factors (TFs) to their target genes and subsequently to enriched biological pathways. Among the 22 identified TF-gene regulatory edges, 9 edges and their associated target genes showed significant enrichment, collectively mapping to 88 pathways (FDR-adjusted *p* < 0.005). The visualization highlights how these regulatory interactions contribute to biological processes relevant to neurodegeneration and Alzheimer’s disease (AD).

To investigate biological processes associated with AD, we performed Gene Set Enrichment Analysis (GSEA) on key transcription factors (TFs) and their target genes. Among the 22 identified TF-gene regulatory edges, 9 TFs and their associated target genes showed significant enrichment, collectively mapping to 88 significantly enriched pathways (FDR-adjusted *p* < 0.005), spanning multiple biological functions relevant to neurodegeneration and AD pathophysiology. Figure 5 shows a Sankey plot illustrating 9 TF-gene edges and their enriched biological pathways. A comprehensive list of enriched pathways identified is provided in Supplementary Data S1.

Pathways associated with synaptic function and neurotransmission, including cholinergic synapse, glutamatergic synapse, GABAergic synapse, synaptic vesicle cycle, and postsynaptic density, were significantly enriched in AD samples. Disruptions in protein homoeostasis and autophagy were also prominent, with ubiquitin-mediated proteolysis, protein processing in the endoplasmic reticulum, mTOR signalling, and autophagy showing enrichment.

GSEA further identified dysregulation in oxidative stress and mitochondrial function-related pathways, including oxidative phosphorylation, mitochondrial matrix components, and transition metal ion binding. Mitochondrial dysfunction and oxidative stress contribute to neuronal damage and energy deficits in AD. Additionally, neuroinflammatory processes and cell adhesion mechanisms were enriched, with pathways such as regulation of actin cytoskeleton, focal adhesion, cell-cell junctions, MAPK signalling, and Ras signalling showing altered activity.

In terms of molecular functions, our analysis identified enrichment in GTPase activity, GTPase binding, guanyl-nucleotide exchange factor activity, protein serine/threonine kinase activity, ubiquitin-protein transferase activity, voltage-gated potassium channel activity, RNA binding, and iron ion binding.

The analysis also revealed enrichment in cellular components, including Golgi membrane, trans-Golgi network, mitochondrial matrix, rough endoplasmic reticulum, nucleus, postsynaptic density, synaptic membrane, neuron projection, and axon guidance.

Additionally, key transcriptional and gene regulatory pathways were identified, including E2F targets, Myc targets V1, histone acetyltransferase activity. Sequence-specific DNA binding, RNA polymerase II transcription regulatory region sequence-specific DNA binding, and transcription cis-regulatory region binding.

### Cell-type relevance highlights the role of immune cells

We conducted a cell-type relevance analysis to investigate the contribution of specific cell populations to AD. We identified ATOH8 as a marker of microglia, NEUROD1 as a marker of neurons, and TCF7 in macrophages, overlapping with the 22 TF-gene edges. This finding shows the involvement of immune response (microglia and macrophages) and neuronal function.

### Correlation analysis with cognitive decline

**Fig. 6:**
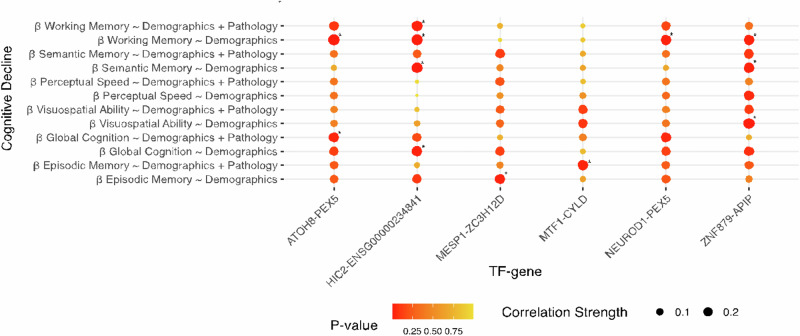
TF-gene edges and cognitive domain measures correlation. Bubble plot depicting the Spearman correlation coefficients and *p*-values (*p* < 0.05) for the association between six significantly correlated TF-gene edges and cognitive domain measures.

To identify potential clinical relevance, we assessed the association between the rates of change of clinical measures and 22 identified TF-gene edges using Spearman correlations. Our findings show that six TF-gene edges are significantly correlated with at least one clinical measure, underscoring their potential clinical importance. Figure 6 shows a bubble plot depicting the Spearman correlation coefficients and corresponding *p*-values (*p* < 0.05) for six TF-gene edges that were significantly associated with cognitive domain measures. Specifically, HIC2-Pseudogene ENSG00000234841 showed significant correlations with semantic memory, global cognition, and working memory. ZNF879-APIP was found to be correlated with semantic memory, visuospatial ability, and working memory, all of which are critical cognitive processes that commonly decline in AD. MESP1-ZC3H12D showed a strong association with episodic memory. ATOH8-PEX5 was correlated with global cognition and working memory, reflecting its importance in cognitive decline, while NEUROD1-PEX5 was specifically tied to working memory, emphasizing its role in memory-related tasks. Moreover, MTF1-CYLD was correlated with episodic memory. These correlations suggest that the identified TF-gene regulatory relationships may be linked to cognitive trajectories observed in AD. Supplementary Fig. S7 shows correlation plots illustrating the relationship between the significant TF-gene edges identified in the regulatory network and rates of cognitive measures.

### Association of identified regulatory TF-gene pairs with previously reported Alzheimer’s disease genes

**Fig. 7:**
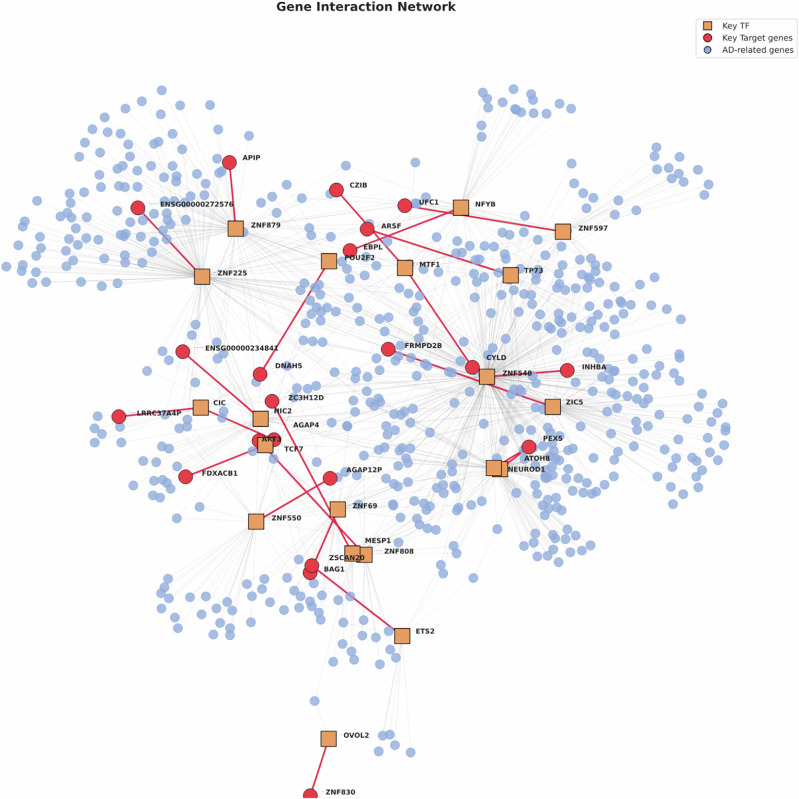
Network of 22 key transcription factors and AD-related target genes. The network visualizes regulatory relationships between the 22 key transcription factors (orange squares) and their associated target genes identified in the gene regulatory networks. Red circles represent key TF target genes forming the 22 TF-gene regulatory pairs, while blue circles represent additional Alzheimer’s disease-related genes curated from the AMP-AD consortium. Red edges highlight the regulatory interactions corresponding to the identified TF-gene pairs, whereas grey edges represent additional regulatory connections between TFs and AD-related genes within the network. This visualization illustrates how the identified TF-gene pairs are embedded within a broader AD-associated regulatory network.

To determine whether our identified 22 key TF-gene pairs regulate other AD-related genes, we examined their association with previously studied AD-targeted genes curated by researchers from the National Institute on Aging’s Accelerating Medicines Partnership in Alzheimer’s Disease (AMP-AD) consortium^14^. We systematically assessed whether these AD-related genes were regulated by the identified TFs from TF-genes in the GRNs. Notably, over half of the AD-targeted genes were regulated by these 22 TF, suggesting a relevance of the 22 TF-genes in AD pathophysiology and in the molecular mechanisms underlying the disease. Figure 7 presents a network plot of the 22 identified transcription factors (TF) and associated AD-targeted genes.

To evaluate whether this overlap could arise by chance, we performed a permutation-based statistical test by randomly selecting TF sets of the same size and repeating the analysis multiple times. The resulting corrected *p*-value was 0.43, indicating that such an overlap could occur under random selection. While the analysis provides contextual evidence suggesting that the identified TFs may influence previously reported AD-related genes, it does not demonstrate statistically significant enrichment.

### Differential expression analysis

Differential expression analysis identified a total of 74 significant DEGs between the AD and NCI samples, using a threshold of adjusted *p*-value (FDR) < 0.05 and absolute log₂ fold change > 0.50. Supplementary Fig. S6A shows a volcano plot of these results, highlighting the significantly Differentially Expressed Genes (DEGs). Among these, only one gene, FRMPD2B, overlapped with key TF-gene edges in the regulatory network. Supplementary Fig. S6B shows a Venn diagram depicting the overlap between the 74 DEGs and the 22 identified TF-gene edges, illustrating the limited intersection. This finding highlights the distinct insights gained from gene regulatory network (GRN) analysis compared to traditional differential expression analysis.

To further investigate the functional relevance of these DEGs, we performed Gene Set Enrichment Analysis (GSEA) using multiple curated gene sets: GO Molecular Function 2023, MSigDB Hallmark 2020, KEGG 2021 (Human), GO Cellular Component 2023. The analysis identified enrichment in key biological processes, including TNF-alpha signalling via NF-kB, structural components like collagen-containing extracellular matrix (GO:0062023) and microvillus (GO:0005902), as well as molecular activities such as hormone activity (GO:0005179) and neutral L-amino acid: sodium symporter activity (GO:0005295).

## Discussion

This study aimed to explore transcriptional network dysregulation in Alzheimer’s Disease (AD) through the analysis of individual-specific gene regulatory network models. Prior work has shown the relevance of GRNs in neurodegenerative disorders, but most analyses rely on population-level networks constructed from aggregated data^15^, which can obscure inter-individual variation, introduce bias, and average out disease-specific regulatory changes. Such approaches make it challenging to identify consistently dysregulated regulatory patterns across individuals and limit the use of rigorous statistical testing to assess their significance. By employing a systems biology approach to model sample-specific genome-scale gene regulatory networks, we addressed this critical gap, uncovering AD-specific regulatory dynamics and identifying transcription factor-gene regulations significantly associated with declines in cognition and memory.

In our analysis, we found significant differences in the regulatory networks between individuals diagnosed with AD and those without cognitive impairment (NCI). Specifically, 22 critical TF-gene interactions reliably distinguished AD from NCI with a weighted classification accuracy of 96%. Among these interactions, ZNF225, ZNF849, and ZNF548 emerged as key hub transcription factors, exerting substantial influence across the networks. Importantly, these 22 key interactions were further validated in independent datasets from the same cohort at single-cell resolution, showing preserved predictive power and confirming their robustness. Additionally, in single-cell validations, two of the three hub TFs (ZNF225 and ZNF548) were consistently identified as central regulatory nodes, highlighting their potential relevance in AD pathology. The prominence of zinc-finger transcription factors as regulatory hubs aligns with existing literature implicating these proteins in neural differentiation, synaptic plasticity, and neurodegeneration^16^.

Further, several of the identified TF-gene interactions, involving the transcription factors NEUROD1 and ATOH8, were significantly correlated with clinical measures of cognitive decline. However, these correlations should be interpreted cautiously, and further experimental validation will be necessary to determine whether these regulatory interactions indeed have mechanistic roles in cognitive decline or potential utility in biomarker development. This approach is particularly informative, as several of the implicated transcription factors, including NEUROD1 and ATOH8, have biologically plausible roles in cognition. NEUROD1 has been associated with hippocampal neurogenesis and memory rescue in AD mice models^17^, and recent evidence shows that AAV-mediated neurod1 gene therapy can induce functional brain repair in Alzheimer’s-like non-human primate models by preventing neuronal degeneration, neuroinflammation, restoring synaptic function, and improving spatial working memory^17^. ATOH8, a member of the atonal-related bHLH transcription factor family, is implicated in neuronal lineage specification and has been shown to regulate postnatal neurogenesis, neural progenitor proliferation, and neuronal differentiation, with loss-of-function studies demonstrating reduced neuronal populations and impaired neurodevelopment. These findings support its potential relevance to neurodegenerative contexts characterized by impaired neurogenesis^18,19^. Thus, specific disruptions in TF-gene regulatory networks appear to have meaningful clinical relevance, suggesting a potential relevance as biomarkers or therapeutic targets.

Moreover, our gene set enrichment analysis identified significant dysregulation in key biological pathways critical to neuronal function in AD, including synaptic transmission, mitochondrial energy metabolism, protein homoeostasis, neuroinflammation, and epigenetic regulation. Synaptic dysfunction was particularly prominent, with disturbances in cholinergic, glutamatergic, and GABAergic pathways, all of which are closely linked to synapse loss and cognitive decline in AD^20–22^. In parallel, our analysis revealed marked mitochondrial dysfunction and oxidative stress, with disruptions in energy metabolism and oxidative phosphorylation leading to decreased ATP production, elevated reactive oxygen species (ROS), and neuronal damage. These findings align with the mitochondrial cascade hypothesis^23^, wherein impaired mitochondrial function amplifies amyloid-β (Aβ) and tau pathology, creating a feedback loop of oxidative damage and synaptic failure that accelerates AD progression. Protein homoeostasis failure also emerged as dysregulated in our analysis, with alterations in critical pathways responsible for protein quality control, including the ubiquitin-proteasome system (UPS), endoplasmic reticulum (ER) protein processing, autophagy, and mTOR signalling. These disruptions are assumed to overwhelm normal protein clearance mechanisms, leading to the accumulation of toxic Aβ plaques and tau tangles^24^. The impairment of proteasomal and autophagic pathways, coupled with hyperactivation of mTOR signalling, contributes to the buildup of amyloid and tau, core drivers of AD pathology^25–27^. Neuroinflammation was another key feature identified in our analysis, with significant upregulation of cytokine signalling, complement activation, and glial markers, reflecting chronic activation of microglia and astrocytes. This persistent neuroinflammation, combined with blood-brain barrier dysfunction, worsens neurodegeneration, as thought to impair Aβ clearance and accelerate AD progression^28–30^. Taken together, these findings are consistent with the concept of “inflammaging,” where chronic, low-grade inflammation in the brain exacerbates neurodegeneration and contributes to AD pathology^30^. Finally, significant transcriptional and epigenetic dysregulation was observed in AD, with alterations in histone acetylation, chromatin organization, and transcription factor binding. Integrated multi-omics studies also confirm the disruption of chromatin architecture and transcriptional regulatory systems, which is consistent with global changes in transcription factor activity. Notably, transcription regulators such as E2F and MYC are among those most significantly affected^14^. E2F1 is significantly elevated in the hippocampus in Alzheimer’s disease models and has been implicated in cognitive decline through activation of NF-κB/GSK-3β signalling, leading to increased oxidative stress and tau hyperphosphorylation^31^. These changes suggest aberrant reactivation of the cell cycle and stress response pathways in neurons, potentially contributing to neurodegeneration. Epigenetic modifications, including chromatin remodelling and disruptions in DNA methylation, exacerbate disease pathology by altering gene expression, which may silence protective genes and activate harmful pathways^32^. Together, these transcriptional regulation shifts highlight the synergistic interplay of multiple mechanisms in AD, including synaptic failure, mitochondrial dysfunction, proteostasis collapse, neuroinflammation, and epigenetic alterations, likely all acting together during the progression of the disease.

Our structural network analysis shows that AD networks undergo topological rewiring rather than a global loss of connectivity. In gene regulatory networks, nestedness measures hierarchical organization, where targets of specialized TFs form subsets of those regulated by highly connected hub TFs. The lower nestedness in AD indicates that broad and specialized regulators are less well coordinated. Although subtle in magnitude, this difference signifies a genuine structural rewiring of TF-target gene overlaps rather than a complete network disruption. Furthermore, the comparable density, average degree, and modularity between groups indicate that the overall volume of interactions and broad functional communities remain preserved. However, the largest component size in AD is much smaller, reflecting a fragmented network. Biologically, this fragmentation might reduce communication among major pathways, compromising the overall robustness of the network against stress.

Several limitations warrant consideration. Our moderate sample size may limit generalizability, and the reliance on postmortem data introduces inherent variability despite rigorous normalization. The cross-sectional nature of post-mortem tissue precludes direct assessments of causality or longitudinal changes in gene regulatory dynamics. Future research is needed to address these limitations by validating identified regulatory interactions in larger independent cohorts and employing longitudinal designs coupled with serial cognitive assessments.

Further mechanistic validation, including experimental modulation of identified hub genes through gene-editing technologies and animal models, will be essential to delineate their precise roles in AD pathology. Moreover, expanding analyses beyond the dorsolateral prefrontal cortex to other vulnerable brain regions such as the hippocampus and entorhinal cortex could provide additional insights. Single-cell and spatial transcriptomic approaches may further refine our understanding of cell-type-specific regulatory changes in AD.

Despite these limitations, the strength of our study lies in keeping individual differences in our analysis by reconstructing sample-specifc Gene Regulatory Networks (GRNs). This approach allows for a deeper understanding of gene regulatory interactions that may be missed in group-based analyses, providing more specific insights into the molecular mechanisms of AD. Additionally, by using GRNs, which model TF-gene interactions rather than relying solely on gene expression values, we focus on the regulatory relationships that drive disease processes, offering a more functional perspective on AD.

In conclusion, this study identifies key transcriptional hubs within sample-specific gene regulatory networks that are significantly associated with cognitive impairment in Alzheimer’s disease (AD). By emphasizing network dysregulation rather than isolated gene alterations, our findings provide new insights into the molecular complexity underlying AD and highlight potential therapeutic targets, including regulatory hubs such as ZNF225, ZNF849, and ZNF548. These results support the hypothesis that AD is a highly complex disease driven by multiple interconnected regulatory interactions that collectively contribute to its molecular pathology. Moreover, our network-based approach extends the insights gained from traditional differential expression analyses by incorporating functional regulatory relationships. This enables the identification of specific transcription factors within patient-specific gene regulatory networks that are directly associated with cognitive decline in Alzheimer’s disease. Overall, this work advances our understanding of the molecular mechanisms of AD and provides a framework for developing future targeted therapeutic strategies.

## Methods

### Ethical approval

All participants enrolled without known dementia and agreed to detailed clinical evaluation and brain donation at death. All studies were approved by an Institutional Review Board of Rush University Medical Center. Each participant signed informed consent and repository consents and all ROSMAP participants signed an Anatomic Gift Act.

### Data acquisition and cohort selection

For the discovery dataset, bulk transcriptomic data from the dorsolateral prefrontal cortex (DLPFC) were obtained from the Religious Orders Study and Memory and Aging Project (ROSMAP)^33,34^, a longitudinal cohort that integrates detailed antemortem cognitive assessments with postmortem neuropathological evaluations to study aging and neurodegeneration.

Cognitive status was determined using the Cogdx consensus diagnosis^35^, based on structured clinical assessments conducted by expert neurologists. Neuropathological classification followed the Consortium to Establish a Registry for Alzheimer’s Disease (CERAD) criteria^36^, which assign AD pathology levels based on semiquantitative assessment of neuritic plaque burden.

To maximize diagnostic specificity and reduce potential confounding, we defined AD cases as participants who met both:

- A clinical diagnosis of AD by cogdx (AD with no alternative cause of CI), and
- A neuropathologic classification of Definite AD by CERAD.

NCI controls were required to have:

- No clinical evidence of cognitive impairment (cogdx = NCI), and
- No neuropathologic evidence of AD (CERAD = No AD).

This combined selection strategy ensured a well-defined case-control structure and reduced the influence of comorbid conditions on transcriptomic analyses.

For validation, we used single-cell transcriptomic data from 48 DLPFC ROSMAP samples collected in a separate study^37^. These samples were filtered and labelled using the same criteria as the discovery dataset, based on Cogdx and CERAD evaluation.

### Data preprocessing

**Fig. 8:**
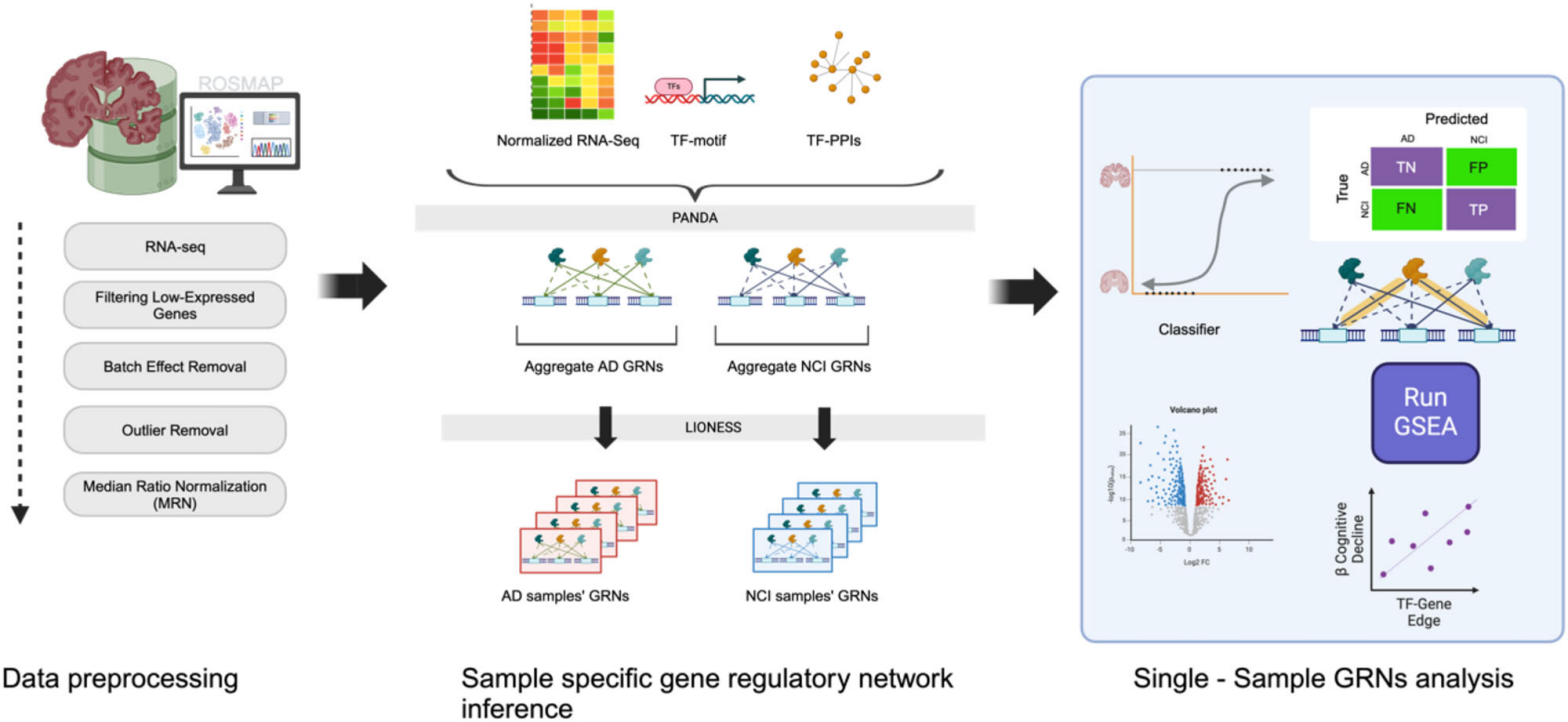
Study overview. The figure presents the stepwise workflow of the integrative analysis pipeline. Step 1 involves data preprocessing, where quality control, normalization, and transformation techniques are applied to ensure data consistency and reliability. Step 2 focuses on constructing sample-specific gene regulatory networks (GRNs) to capture transcription factor (TF)-gene interactions unique to each sample or condition. Step 3 involves identifying key TF-gene regulatory edges based on network topology and statistical significance. Gene set enrichment analysis is then conducted to reveal biologically relevant pathways and functional categories associated with the identified regulatory interactions. Additionally, correlation analysis between TF-gene interactions and cognitive decline measures, along with differential expression analysis, is performed to gain deeper insights into the underlying molecular mechanisms.

An overview of the analytical workflow used in this study is presented in Fig. 8, which summarizes the main steps including data preprocessing, gene regulatory network reconstruction, identification of key regulatory interactions, and downstream functional analyses.

In the discovery bulk transcriptomics dataset, we applied a count-per-million (CPM) based expression filter to remove low-expressed genes to retain only biologically relevant genes for analysis. The filtering was performed separately within the AD and NCI groups, retaining genes with CPM ≥ 1 in at least 50% of samples in each group. The final gene set was defined as the union of genes passing this threshold in either group, ensuring that genes expressed in a high proportion of AD or NCI samples were retained for downstream analyses.

To assess and correct for potential batch effects, we conducted an ANOVA test on principal component analysis (PCA) components to determine whether batch-associated variability was significant. To address the identified batch effects, we applied Combat-Seq^12^ for batch correction. However, batch effects remained significant even after correction; thus, we excluded batches with persistent effects on gene expression despite Combat-Seq correction.

Additionally, to refine RNA-Seq expression data and minimize the influence of extreme values, we applied quantile-based filtering (5th and 95th percentiles) on PCA components, removing samples with outlier expression patterns. This approach improved dataset integrity by reducing the impact of aberrant values that could skew downstream analyses.

Finally, to address technical biases and ensure comparability across samples, we implemented Median Ratio Normalization (MRN) on raw counts.

On validation single-cell transcriptomic data, we generated pseudobulk counts by aggregating all cells together without considering cell type annotations. The pseudobulk counts were then converted to CPM. These CPM normalized pseudobulk expression profiles allow us to compare regulatory patterns identified in the bulk transcriptomic data.

### Gene regulatory network modelling

To construct aggregate gene regulatory networks (GRNs) as a basis to infer sample-specific GRNs for Alzheimer’s disease (AD) and no cognitive impairment (NCI), we employed PANDA (Passing Attributes between Networks for Data Assimilation)^10^ on all genes that passed our expression filtering criteria of preprocessing. PANDA integrates multiple data modalities (gene expression data, a prior transcription factor (TF)-motif data, and protein-protein interactions (PPIs)) to infer regulatory networks. For this study, PPIs were sourced from STRING^38^ with a confidence threshold of 0.5, while the initial TF-motif prior was generated using the FIMO tool^39^ to map motifs from JASPAR^40^ across target gene promoters. PANDA refines these TF-target gene interactions via an iterative message-passing algorithm that updates edge weights based on responsibility (TF cooperativity) and availability (gene co-expression) using Tanimoto-based similarity. The resulting TF-target link represents a weighted probability that a TF functionally regulates a specific gene.

To assess inter-individual variability in transcriptional regulation, we applied LIONESS^11^ to reconstruct sample-specific GRNs from aggregate networks generated by PANDA. This approach estimates individual TF-gene interactions, with edge weights reflecting transcriptional regulation strength.

To account for disease-specific regulatory patterns, we derived sample AD sample-specific GRNs from the AD aggregate network and NCI sample-specific GRNs from the NCI aggregate network. By constructing these sample-specific gene regulatory networks, we aimed to capture inter-individual heterogeneity, reflecting individual differences in transcriptional regulation that may underlie individual disease characteristics, disease susceptibility, and trajectory.

To increase the sensitivity of downstream analyses and ensure the robustness of the detected regulatory mechanisms, we retained the top 5% of edges based on their weights for both NCI and AD in the discovery bulk RNA-Seq dataset. Prioritizing robustness allows the selection of regulatory interactions that are consistently supported by the data, which is particularly important given the complexity and high dimensionality of transcriptomic datasets. This stringent filtering reduces the likelihood of false-positive findings arising from technical noise, enabling the identification of the most stable and reproducible regulatory interactions. After applying these thresholds, we compared the resulting GRNs to identify regulatory interactions that were common to both conditions as well as those that were condition-specific. Downstream analyses were then performed on both the common edges and the condition-specific edges. For condition-specific edges, we extracted the corresponding edge weights from the top 5% of GRNs retained for each condition, considering only edges present exclusively in one condition. To ensure that the sample-specific GRNs accurately reflect true biological variation rather than technical noise or sample-specific artifacts, we validated the top-weighted regulatory edges by assessing whether the inferred transcription factors (TFs) had known binding motifs in the regulatory regions of their target genes. This approach could confirm that the strongest predicted regulatory interactions are supported by biologically plausible TF-target relationships and provide confidence that the inferred sample-specific GRNs capture meaningful regulatory dynamics rather than reflecting technical variation in the data.

For the single-cell transcriptomic data used for validation, we followed a workflow analogous to the one used for the discovery bulk RNA-Seq dataset to reconstruct sample-specific GRNs.

### Network analysis of AD-specific and NCI-specific GRNs

To analyze the structural properties and identify potential differences in AD-specific and NCI-specific GRNs, we calculated key network metrics for both AD-specific and NCI-specific GRNs on the top 5% retained edges. The metrics included density, which measures the proportion of actual connections to possible connections in the network; average degree, representing the average number of connections per node; modularity, quantifying the extent to which the network can be divided into distinct communities; largest component size, indicating the size of the largest connected subgraph; and nestedness, assessing the hierarchical structure of the network. These metrics were calculated to provide insights into the connectivity, robustness, and organization of the regulatory networks, helping to reveal how TF-gene interactions differ in the context of AD and NCI based on network structure.

### Identification of differentially regulated transcription factor-target gene pairs

To identify differentially regulated target genes and their associated transcription factors (TFs), we employed logistic regression^41^ on TF-target Genes’ edge weights of gene regulatory networks (GRNs) of the discovery dataset to classify AD and NCI samples. To select the most informative features for classification, we applied Recursive Feature Elimination (RFE)^42^ using logistic regression with default hyperparameters on the training portion of a random train-test split, which iteratively removes half of the features based on model-derived feature importance.

Following feature ranking by RFE, logistic regression models were trained iteratively on the same random train-test split using the top-ranked features in descending order of importance. Starting from the highest-ranked features, additional features were progressively included, and model performance was evaluated at each step. The optimal feature set was determined at the point where classification accuracy reached its maximum before beginning to decline.

To obtain a robust estimate of model performance on the discovery dataset, Monte Carlo cross-validation with 100 iterations was performed on the discovery bulk RNA-Seq dataset using the key selected features. The mean accuracy, F1 score, and AUC reported represent the average Monte Carlo cross-validated performance. We further assessed TF-target gene co-expression patterns by calculating the Pearson correlation between each key TF and its target gene across all AD and NCI samples in the discovery dataset, in order to determine whether these interactions were differentially co-expressed in both AD and NCI groups, thereby validating our findings.

To assess the statistical significance of differentially regulated edges on the discovery bulk RNA-Seq dataset, we performed a bootstrapping analysis, randomly selecting 22, 100, 500, and 1000 TF-target gene pairs and conducting 1000 iterations for each subset. For each iteration, we calculated classification accuracy, ensuring that the identified regulatory interactions were robust and non-random.

In addition to analyses based on common edges, we also performed differential analyses using condition-specific edges (edges present only in either the AD or NCI network). For this analysis, model performance was evaluated using Monte Carlo cross-validation with 100 iterations, and the mean f1, auc and classification accuracy were calculated.

### Validation of differentially regulated transcription factor-target gene pairs

To validate the key differentially regulated TF-target gene pairs identified in the discovery dataset, we trained logistic regression models on the sample-specific GRNs inferred from the single-cell validation dataset, using the edges identified from the discovery sample-specific GRNs. Monte Carlo validation with 100 iterations was performed to assess model performance and predictability of key TF-target genes on validation data.

### Pathway and cell type-specific enrichment analysis

To investigate the functional relevance of key transcription factors (TFs) and their target genes within gene regulatory networks (GRNs), we performed Gene Set Enrichment Analysis (GSEA) on each key TF-gene pair and its TF’s immediate network neighbours from the aggregated GRN. This approach integrates each key TF with its direct regulatory targets to capture its local network context. GSEA was conducted using the GSEAPY Python package^43^, with gene sets sourced from the GO Molecular Function 2023, GO Cellular Component 2023, KEGG 2021 (Human), and MSigDB Hallmark 2020 MSigDB Hallmark 2020 databases^44,45^.

To assess the cell-type relevance of identified TFs and genes, we compared them with known markers of distinct neuronal and glial populations from CellMarker 2.0^46^, a curated repository integrating data from over 100,000 publications on human and mouse tissues. This analysis enabled us to link regulatory interactions to specific brain cell types, providing additional insight into the cellular context of AD-associated transcriptional dysregulation.

### Identification of Hub TF

Hub transcription factors (TFs) were identified based on their connectivity within the GRNs. A TF was considered a hub if its connectivity, measured as the number of downstream targets, was at least twice the average degree of all transcription factors in the network. This criterion highlighted TFs with disproportionate regulatory influence within the network.

### Correlation analysis with clinical measures

To assess whether the identified regulatory edges are significantly associated with clinical measures of cognitive decline, Spearman correlation coefficients were calculated between the edge weights of the identified TF-gene interactions in the Gene Regulatory Networks (GRNs) and the estimated rates of change in various cognitive domains over time. These domains included global cognition, episodic memory, working memory, semantic memory, perceptual speed, and visuospatial ability, with the rates of change derived from regression models used in a previous study^47^ that controlled for demographics or both demographics and pathology. Specifically, the following regression models were used: β Global Cognition ~ Demographics, β Global Cognition ~ Demographics + Pathology; β Episodic Memory ~ Demographics, β Episodic Memory ~ Demographics + Pathology; β Working Memory ~ Demographics, β Working Memory ~ Demographics + Pathology; β Semantic Memory ~ Demographics, β Semantic Memory ~ Demographics + Pathology; β Perceptual Speed ~ Demographics, β Perceptual Speed ~ Demographics + Pathology; and β Visuospatial Ability ~ Demographics, β Visuospatial Ability ~ Demographics + Pathology.

By assessing the strength and direction of these correlations, we aimed to find potential relationships between specific TF-gene interactions and the progression of cognitive impairment.

### Differential expression analysis between AD and NCI

To identify key differentially expressed genes (DEGs) and assess their relationship to disease-specific regulatory interactions within the gene networks, we performed differential gene expression analysis between AD and NCI samples. Using pyDESeq2^48^, which implements the DESeq2 methodology within a Python framework, we normalized the expression data with Median Ratio Normalization (MRN) and modelled it using a negative binomial distribution to account for sample variability. Differential expression was assessed using a likelihood ratio test, with significance determined by an adjusted *p*-value (FDR) < 0.05 and a log₂ fold change > 0.50.

## Supplementary information

Supplementary Information

Supplementary Data S1

Supplementary Data S2

## Data Availability

RNA-Seq data**:** https://www.synapse.org/Synapse:syn3219045 The datasets generated during this study are available in the Zenodo repository: 10.5281/zenodo.19543885.

## Code availability

GitHub: https://github.com/Polster-lab/Analysis_GRN_ROSMAP,.
