# Supplementary material for: Network-based discovery of regulatory drivers of cognitive decline in alzheimer’s disease
Source: NPJ Aging. 2026 Jul 16;12(1):95. doi: 10.1038/s41514-026-00443-0 (PMC13376175; doi:10.1038/s41514-026-00443-0)
Supplement: Supplementary file 1 — Supplementary Information [file 41514_2026_443_MOESM1_ESM.pdf]

Supplementary Figures

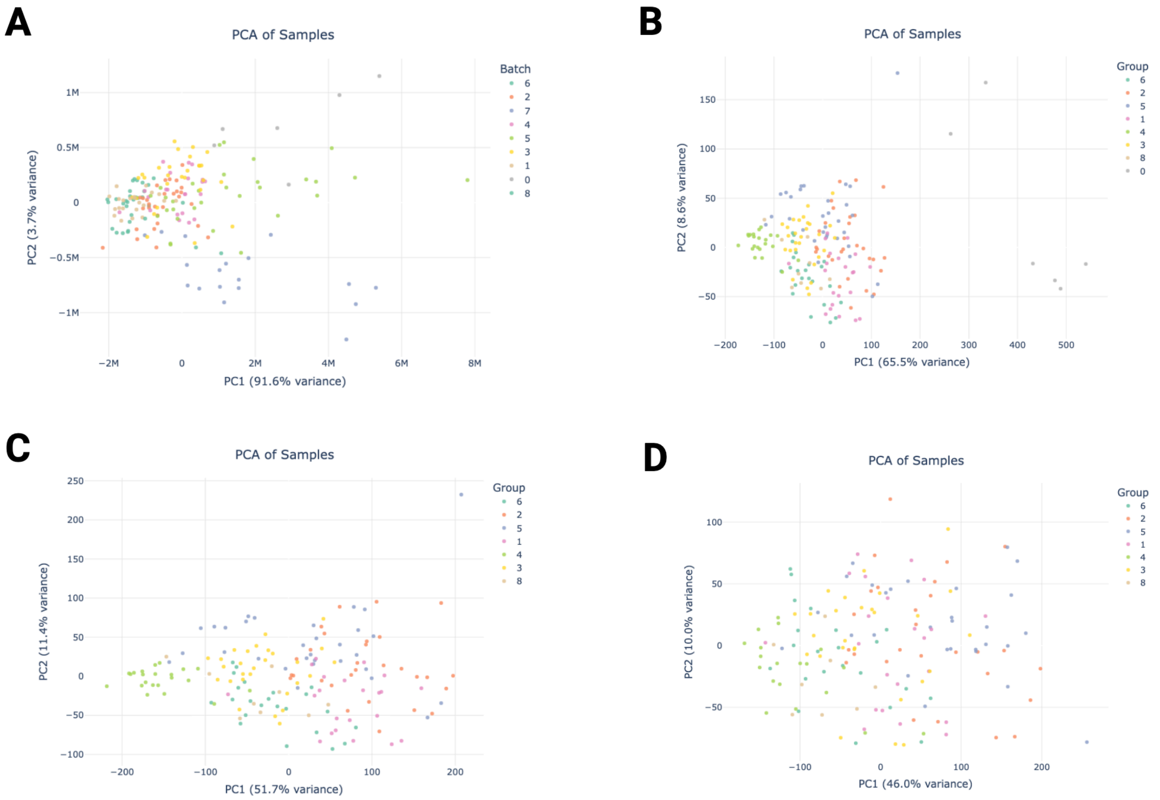

Figure S1. Principal component analysis (PCA) illustrating sample filtering and batch correction. Samples are color-coded by batch, with each color representing a distinct batch.

A. PCA of the original dataset after CPM threshold filtering (17,330 genes)

B. PCA after removing batch 7 (97 AD, 73 NCI samples)

C. PCA after removing batch 0 (94 AD, 70 NCI samples)

D. PCA after quantile-based outlier removal (87 AD, 67 NCI samples)

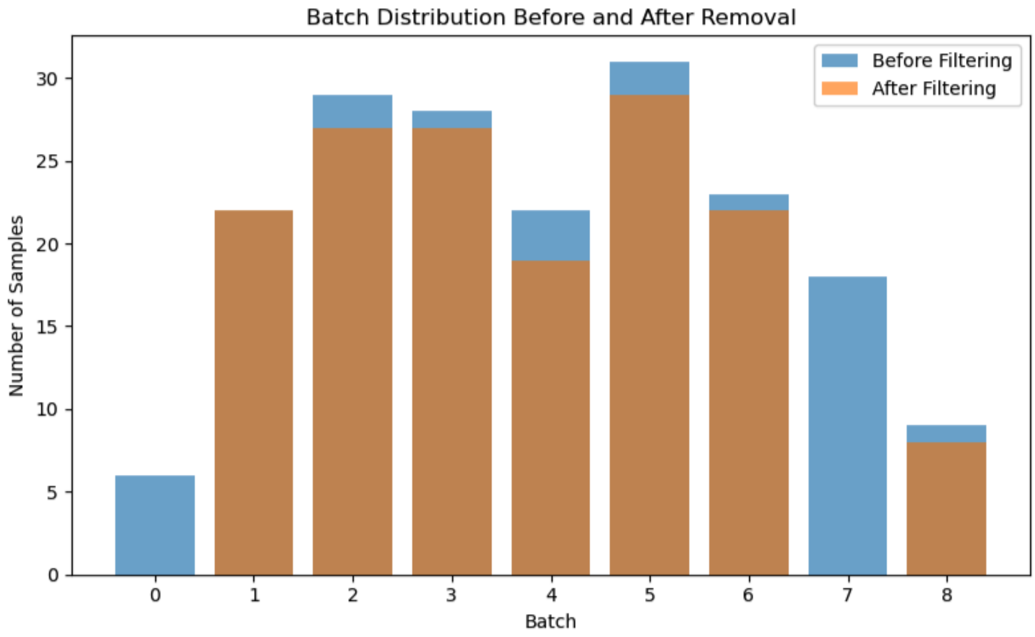

Figure S2. Distribution of Samples Across Batches Before and After Filtering

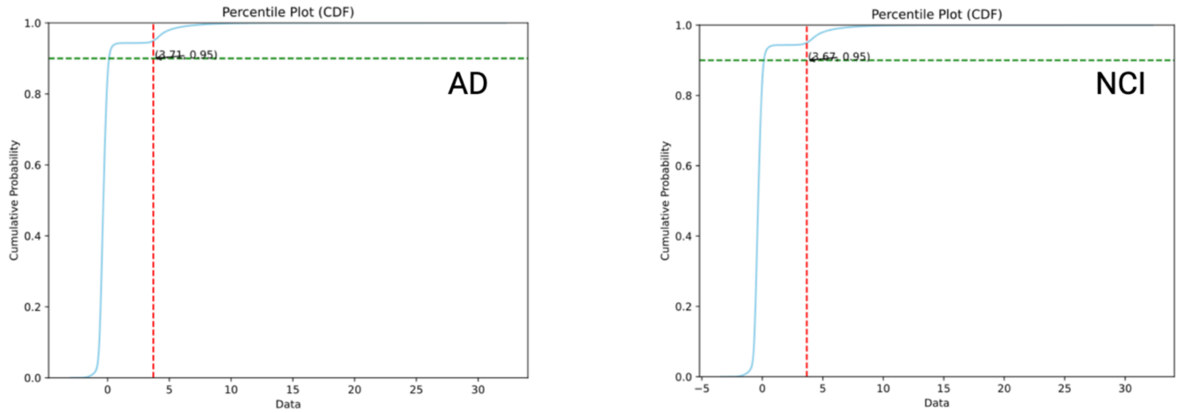

Figure S3. Cumulative distribution of edge weights for Alzheimer's Disease (AD) and Mild Cognitive Impairment (NCI) networks, highlighting the threshold selection for the top 5% of regulatory interactions

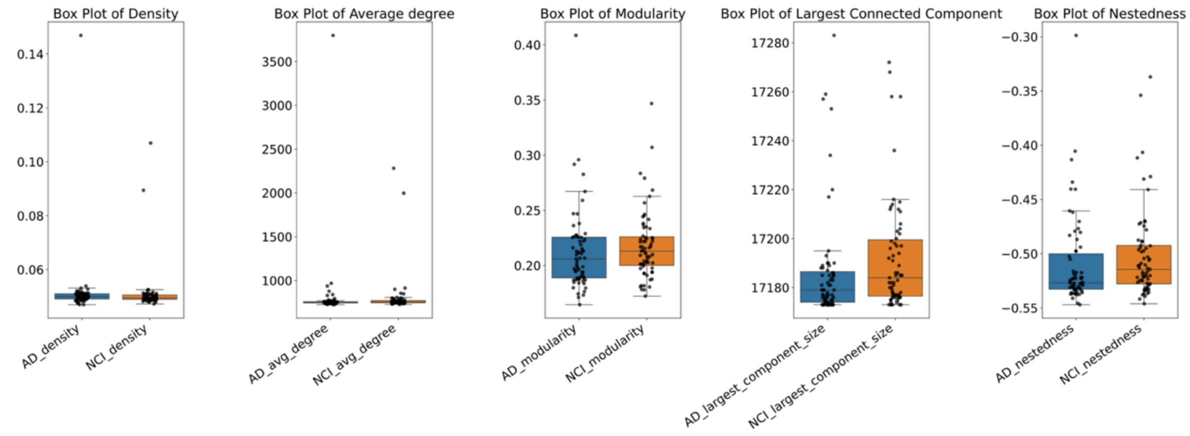

Figure S4. Box plots showing network metrics (density, average degree, modularity, giant component size, and nestedness) in Alzheimer's Disease (AD) and No Cognitive Impairment (NCI) networks

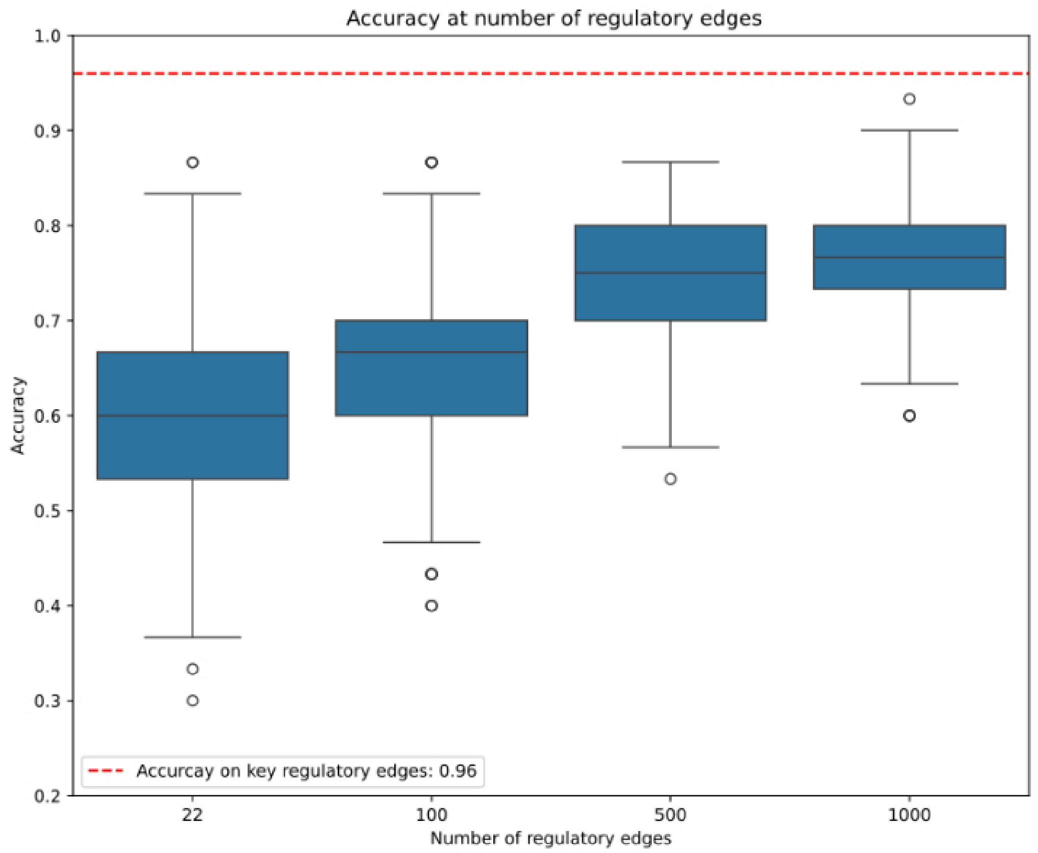

Figure S5. Box plots showing the classification accuracy at different levels of random TF-target gene pair selection (22, 100, 500, and 1000 gene pairs) across 1000 bootstrapping iterations

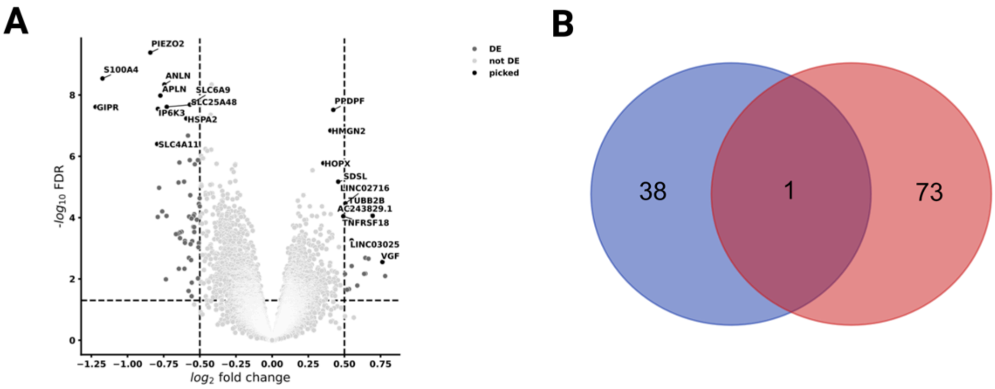

Figure S6. Differential Expression Analysis on AD vs NCI
A. Volcano plot of differential expression analysis highlighting significantly differentially expressed genes (DEGs)

B. Venn diagram showing the overlap between DEGs and the 22 identified TF-gene edges, illustrating the intersection of key regulatory interactions with differentially expressed genes

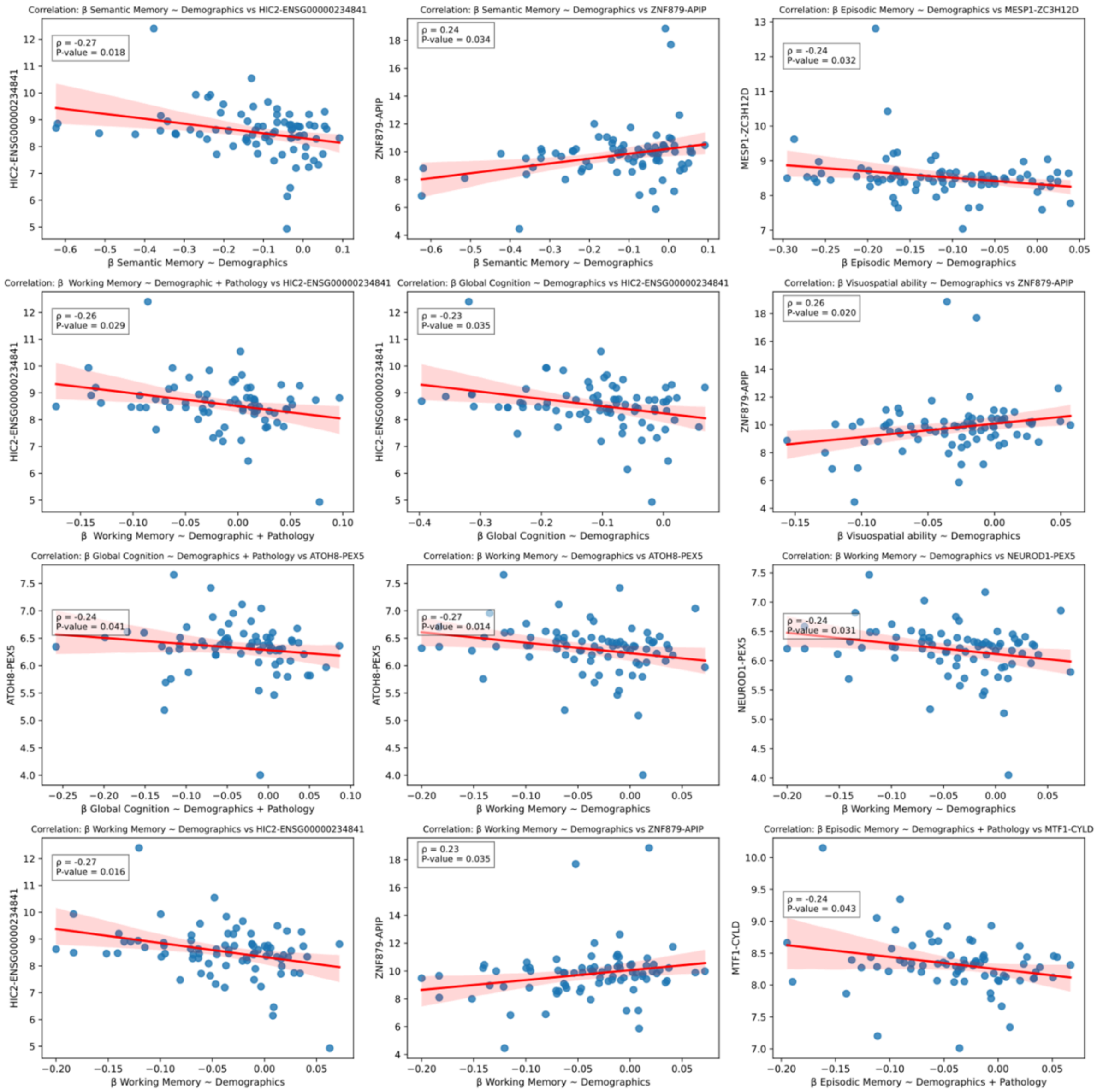

Figure S7. Correlation plots showing the relationship between significant TF-gene edges and rates of cognitive measures

Supplementary Tables

Supplementary Data S1. Enriched Pathways (p-adjusted value <= 0.05) corresponding to 22 key identified edges

Batch	Raw_Cohort_Count	Final_Cohort_Count
5	31	29
2	29	27
3	28	27
6	23	22
1	22	22
4	22	19
7	18	0
8	9	8
0	6	0

Supplementary Data S2. Distribution of samples across sequencing batches before and after batch exclusion in the AD and NCI cohorts.
